# Identifying Problematic Internet Users: Development and Validation of the Internet Motive Questionnaire for Adolescents (IMQ-A)

**DOI:** 10.2196/jmir.3398

**Published:** 2014-10-09

**Authors:** Christina Bischof-Kastner, Emmanuel Kuntsche, Jörg Wolstein

**Affiliations:** ^1^Institute of PsychologyFaculty of HumanitiesUniversity of BambergBambergGermany; ^2^Addiction SwitzerlandLausanneSwitzerland

**Keywords:** Internet, adolescents, questionnaires, validation, addictive behavior, statistical factor analysis

## Abstract

**Background:**

Internationally, up to 15.1% of intensive Internet use among adolescents is dysfunctional. To provide a basis for early intervention and preventive measures, understanding the motives behind intensive Internet use is important.

**Objective:**

This study aims to develop a questionnaire, the Internet Motive Questionnaire for Adolescents (IMQ-A), as a theory-based measurement for identifying the underlying motives for high-risk Internet use. More precisely, the aim was to confirm the 4-factor structure (ie, social, enhancement, coping, and conformity motives) as well as its construct and concurrent validity. Another aim was to identify the motivational differences between high-risk and low-risk Internet users.

**Methods:**

A sample of 101 German adolescents (female: 52.5%, 53/101; age: mean 15.9, SD 1.3 years) was recruited. High-risk users (n=47) and low-risk users (n=54) were identified based on a screening measure for online addiction behavior in children and adolescents (Online-Suchtverhalten-Skala, OSV_K_-S). Here, “high-risk” Internet use means use that exceeds the level of intensive Internet use (OSV_K_-S sum score ≥7).

**Results:**

The confirmatory factor analysis confirmed the IMQ-A’s 4-factor structure. A reliability analysis revealed good internal consistencies of the subscales (.71 up to .86). Moreover, regression analyses confirmed that the enhancement and coping motive groups significantly predicted high-risk Internet consumption and the OSV_K_-S sum score. A mixed-model ANOVA confirmed that adolescents mainly access the Internet for social motives, followed by enhancement and coping motives, and that high-risk users access the Internet more frequently for coping and enhancement motives than low-risk users. Low-risk users were primarily motivated socially.

**Conclusions:**

The IMQ-A enables the assessment of motives related to adolescent Internet use and thus the identification of populations at risk. The questionnaire enables the development of preventive measures or early intervention programs, especially dealing with internal motives of Internet consumption.

## Introduction

Internet use among so called “digital natives” [1,2] is linked to all areas of life [3-7]. For example, every German household containing young people aged between 12 and 19 years is equipped with a computer or laptop [5]. In addition, personal computers are no longer the most common way of accessing the Internet in Europe. There has been unequivocal growth in access to the Internet via handheld or portable devices (eg, touchpads and smartphones), showing that the Internet is now accessible to everyone [3,5]. Therefore, we can assume the existence of a generation that has grown up with the latest technologies from a very young age [1,2] and that Internet use is an extremely widespread phenomenon. This situation can be clearly explained by the fact that the Internet is a convenient source of information, social contacts, education, shopping, and recreational activities [6-10] that simplifies everyday life.

The Internet also has a negative side. The inclusion of Internet Gaming Disorder in Section III of the *Diagnostic and Statistical Manual of Mental Disorders* (Fifth Edition) (*DSM-5*) [11] illustrates the relevance of Internet addiction. Furthermore, different studies in several countries show that for 1.2% to 15.1% of cases, the intensive Internet use of adolescents can be classified as problematic [12-17]. Initial results from longitudinal studies even give rise to the suspicion that the disorder is highly stable [18].

To provide a basis for early intervention and preventive measures, we need to understand how the frequent Internet use of approximately 8% of adolescents becomes a problematic or dysfunctional behavior. Regarding the relationship between the Internet and all areas of life [5-7] and the suggested *DSM-5* criteria for Internet addiction [11], the duration someone spends online does not appear to be a valid criterion. Thus, getting to know the motives behind adolescent Internet consumption is important.

Concerning motives for media use in general, McQuail [19,20] assumes 4 basic motives: information, personal identity, integration and social identity, and entertainment, the latter covering relaxation, emotional relief, recreation, and more. Recent research regarding the motivations of Internet use in particular found the existence of instrumental motives, such as information seeking and social interaction, as well as a relationship between personality types and Internet use [6,21-28]. For example, Amiel and Sargent developed the Internet Motives Questionnaire [21], which measures the 4 instrumental motive groups interpersonal/communication utility, entertainment utility, information utility, and convenience.

There are, however, a number of gaps in current research. First, not much is known about affectivity, which plays an important role in understanding problematic Internet use. In fact, there are instruments for measuring Internet addiction and instrumental motives for using the Internet. In 1998, for example, Young [29] developed the first instrument for measuring Internet addiction, the Internet Addiction Test (IAT), which was translated into various languages and validated in different samples [30-33]. Also, the previously mentioned Internet Motives Questionnaire assesses motives that are more instrumental as they target the affective function the Internet serves for adolescents.

According to the motivation model of McClelland [34], affective change is assumed to be the driving force behind human behavior. Hence, striving for positive affect and escaping or relieving a dysphoric mood is seen as the basis of motivational (Internet) behavior and thus, according to the *DSM-5*, a criterion for Internet use disorder [11].

Second, none of the studies were based on a theoretical framework defining the dimensionality of motives for Internet use a priori. One exception is the Motives for Online Gaming Questionnaire (MOGQ) [35]. The MOGQ is based on a theoretical approach, although this model was developed by observing online players. Thus, the questionnaire is specific to Internet gaming and, therefore, not applicable to Internet use motives in general.

One promising approach that includes affectivity and allows the classification of the motivation behind human behavior was proposed by Cox and Klinger [36,37]. The Motivational Model of Alcohol Use assumes that people display a certain behavior to achieve expected or desired effects [38-42]. Motives can be classified by means of the valence and the source of the expected affective change. The valence is either positive (ie, to increase positive feelings) or negative (ie, to decrease negative feelings); the source can be internal (eg, in respect to one’s own bodily sensations) or external (eg, in respect to significant others). Crossing these 2 dimensions results in the 4 motive categories enhancement, coping, conformity, and social as shown in Table 1 [40-44].

**Table 1:** Classification of drinking motives based on the Motivational Model of Alcohol Use by Cox and Klinger [36,37].

Source	Positive valence	Negative valence
Internal	Enhancement motives	Coping motives
External	Social motives	Conformity motives

Based on this 4-dimensional model, Cooper [38] developed the Drinking Motive Questionnaire (DMQ-R; German, French, and Italian version by Kuntsche et al [39]), which comprises 5 items per dimension. Although originally developed to understand alcohol use, the DMQ-R with its 4 motivational factors has already been successfully adapted to several behaviors beyond alcohol use (eg, gambling [41], sexual risk-taking behavior [42], and listening to music [43]). Given the success of previous adaptation attempts, the fact that McQuail’s [19,20] basic motives for using media in general are reflected in the 4-dimensional model of Cooper [38] and the current state of research that assumes social interactions and conformity as motives for Internet consumption (as described previously), it is likely that Cox and Klinger’s motivational model can be applied to Internet use.

Consequently, based on the DMQ-R and the motivational model by Cox and Klinger [36,37], the aim of this study is to develop the Internet Motive Questionnaire for Adolescents (IMQ-A) (see Multimedia Appendices 1 and 2) as a theory-driven conceptualization and to validate it in a sample of German adolescents in a Web-based data collection conducted in the summer of 2011. More specifically, the following hypotheses are tested: (1) The IMQ-A 4-factor model has an adequate fit, (2) every subscale of the IMQ-A (coping, enhancement, social, and conformity motives) has at least satisfactory internal consistency, (3) based on evidence from studies on alcohol use [38,39] and gambling [41], we expect enhancement and coping to be associated with high-score Internet use, but not social or conformity motives, and (4) problematic and nonproblematic Internet users differ with regards to their motives for going online.

## Methods

### Pretest

To examine the usefulness and quality of the IMQ-A, 70 students (female: 71%, 50/70; age: mean 15.90, SD 0.89 years) from 2 schools in Bamberg (Bavaria, Germany) completed a paper-and-pencil questionnaire in January 2011. The students had to answer the 20 items of the initial IMQ-A as well as questions concerning the comprehensibility of the items and the usefulness of the questionnaire. This allowed duration, comprehensibility of the content, and linguistic matching with the target group to be examined. Following the evaluation, particular items were adapted based on feedback from the students.

### Study Design

Data were collected in a Web-based survey from June to September 2011 using the free software package onlineFragebogen [45]. Online questionnaires are usually characterized by good acceptance among adolescents, high data quality, and external validity, particularly when participants complete the questionnaire voluntarily and without temporal constraint [46,47].

The participants were recruited in the German districts of Thuringia, Saxony, and Bavaria through posters, a press release, Facebook, various sports and youth clubs in Bamberg (Bavaria), and broadcasts by local radio stations. Interested individuals were included in a mailing list and provided with an automatically generated personal link required to log in to the study’s website. Strict confidentiality and anonymity were guaranteed. Participants had to answer every question before proceeding to the next page.

After completing all 8 pages, adolescents wishing to take part in a random prize draw for a chance to win 1 of 3 Apple iPod Shuffles were invited to enter their email address. The email address was saved separately from the information collected in the questionnaire.

### Participants

In total, 107 adolescents participated in the study. However, 6 participants were excluded because they were outside of the defined age range (14 to 19 years). The final sample consisted of 101 adolescents (age: mean 15.85, SD 1.33 years). Characteristics of the sample are shown in Table 2.

**Table 2:** Sample information concerning demographics and general aspects of Internet use (N=107).

Characteristics with prevailing response options		n (%)
**Sex**		
	Female	53 (52.2)
	Male	48 (47.8)
**Occupation**		
	Student	88 (87.1)
	Trainee	6 (5.9)
	Job-hunting/apprenticeship-hunting	4 (4.0)
	Student at university	2 (2.0)
	Employed part-time	1 (1.0)
**Type of school/ educational qualifications**		
	High school	62 (61.4)
	Secondary/junior high school	38 (37.6)
	Other	1 (1.0)
**Type of Internet access** ^a^		
	Own computer/laptop	80 (79.2)
	Web-enabled mobile phone	34 (33.7)
	Parental computer/laptop	21 (20.8)
	School computer/laptop	12 (11.9)
**Parental control of Internet use**		
	Yes	18 (17.8)
	No	83 (82.2)
**Online frequency**		
	Daily	90 (89.1)
	2-3 times per week	10 (9.9)
	Once a week	1 (1.0)
**Online activities** ^a^ **(very often or often)**		
	Entertainment (eg, music, videos, pictures)	96 (95.1)
	Online communities (eg, Facebook)	94 (93.1)
	Information research	86 (85.1)
	Messenger (eg, ICQ, Skype)	59 (58.4)
	Email contact	53 (52.5)
	Chatting (eg, chat forums)	47 (46.6)
	Shopping	21 (20.8)
	Online games (eg, Wow, strategy games)	20 (19.8)
	Online banking	4 (4.0)
	Online gambling	0 (0.0)
	Online sex offers	0 (0.0)

^a^ Multiple answers possible.

### Measures

#### Demographics and General Aspects of Internet Use

This questionnaire contains information about age and the characteristics mentioned in Table 2.

#### Screening Measure for Online Addiction Behavior in Children and Adolescents

The Online-Suchtverhalten-Skala (OSV_K_-S) [48] (also Wölfling K, Müller KW, Beutel ME, unpublished data, 2009) used in this study is a German self-report measure for recording the addiction-like use of various Internet applications. This questionnaire was designed based on the screening scale for computer gaming behavior (Screener zum Computerspielverhalten; CSV-R) [49] and the international classification criteria (*DSM-IV* [50]; ICD-10 [51]) for substance addictions [9] (also Wölfling K et al, unpublished data, 2009). The 16 items of the diagnostic module inquire about usage frequency, potential negative consequences of Internet use and pathological consumption patterns. With the help of 14 of these diagnostic items, a score can be computed that classifies the online behavior into the categories low-key, intensive, problematic/abusive, and addicted/pathological. In this case, however, only a subdivision into high-risk and low-risk Internet users was used. Here the risk for being prone to Internet addiction is meant. Therefore, in each case 2 categories were rolled into 1 (low-key and intensive to low-risk; problematic and addicted to high risk). Consequently, a cut-off point of approximately 7 points was used for group assignment.

Owing to the satisfactory to excellent results for psychometric characteristics, such as reliability (α=.88), construct and factorial validity and clinical usefulness, the OSV_K_-S is a promising instrument for Germany [52] (also Wölfling K et al, unpublished data, 2009).

#### Internet Motive Questionnaire for Adolescents

Based on the Drinking Motive Questionnaire Revised (DMQ-R) [38,39], the introduction is worded as follows: “Think of all the times you have been online during the last 12 months; how often do you go online...” Subsequently, 20 items measuring 4 dimensions were presented. Assuming the transferability of the statements, 12 items were taken from the DMQ-R without rewording (eg, “to forget your worries”); 8 items were adapted with regard to Internet use (eg, “because it improves parties and celebrations” was changed to “to improve contact with friends and acquaintances”). Each of the 5 items per dimension (ie, enhancement, coping, social, and conformity) were rated on a 5-point relative frequency scale with answer categories ranging from “(almost) never” (coded as 1) to “(almost) always” (coded as 5).

### Statistical Analyses

Because some of the items of IMQ-A were adapted from a drinking motive questionnaire, we first conducted an explorative factor analysis using SPSS version 20 (IBM Corp, Armonk, NY, USA).

Confirmatory factor analysis (CFA) was used to confirm the 4-factor structure and construct validity of the 16-item IMQ-A. The CFA was performed with SPSS Amos version 20 using the Bollen-Stine bootstrap correction to account for nonnormal distribution [53]. Errors were allowed to correlate. To evaluate the model fit, the fit indices chi-square divided by degrees of freedom (reduced chi-square, χ^2^_red_), comparative fit index (CFI), Tucker-Lewis index (TLI), root mean square error of approximation (RMSEA), and standardized root mean squared residual (SRMR) were used. The parsimony measurement χ^2^_red_ suggests an acceptable fit with values between 1 and 2 [54], whereas CFI and TLI should show values greater than .90 [55]. Regarding RMSEA and SRMR, results lower than .10 were sought; values ranging from .05 to .08 were treated as acceptable fit, and figures between .08 and .10 as moderate fit [55,56].

Cronbach alpha values were used as a measure of internal consistency for which values greater or equal to .9, .8, and .7 are considered as excellent, good, and acceptable, respectively [53,57].

To test concurrent validity of the IMQ-A, multiple regression was performed with the 4 motive groups as independent variables. Due to the dichotomy of the dependent variable high-risk Internet use, a logistic regression was performed. In the second model, a multiple linear regression, the OSV_K_-S sum score was the dependent variable.

Differences in the 4 motive dimensions in the entire sample and among high-risk and low-risk users were tested using a 2 (high-risk vs low-risk users) × 4 (motive dimension) mixed-model analysis of variance (ANOVA). Whenever an overall effect or an interaction was significant, post hoc tests (Bonferroni) were conducted to determine whether high-risk or low-risk users differed on a motive dimension. Descriptive analyses and the mixed-model ANOVA were performed using the SPSS 20.0 statistical software package.

## Results

### Descriptive Statistics

Descriptive results show that 47 of 107 participants (46.5%) were identified as high-risk users and 54 (53.5%) as low-risk users. The groups did not differ significantly in age, but did in gender and education (Table 3). Most high-risk users were male (32/47, 68%), and high school and secondary school students were equally represented (18/47, 38% each). By contrast, the low-risk users were mostly female (38/54, 70%) and grammar school students (44/54, 81%). Significant differences between the 2 groups were also found in terms of variables regarding the Internet behavior of the respondents (eg, online duration) (Table 3).

Although no significant differences emerged regarding the duration of use on weekends, online frequency, and parental control, a higher online duration on weekdays was reported by high-risk users. This pattern also became apparent when examining the regular online duration (high risk: mean 2.63, SD 1.61 hours; low risk: mean 1.77, SD 0.96 hours).

**Table 3:** Sociodemographic- and consumption-relevant characteristics of low-risk and high-risk adolescent Internet users (N=107).

Characteristics	Low risk (n=54)	High risk (n=47)	χ^2^_1_	*t*_99_	*U*	*P*
Male, n (%)	16 (29.6)	32 (68.1)	14.9			<.001
Parental control, n (%)	8 (14.8)	10 (21.3)	0.7			.40
Age, mean (SD)	15.94 (1.31)	15.74 (1.36)		0.752		.45
Internet use on weekdays (in hours), mean (SD)	2.63 (2.40)	5.00 (4.14)		–2.685		.009
Internet use at the weekend/on holidays (in hours), mean (SD)	3.37 (1.83)	3.68 (2.60)		–0.482		.63
Education, mean rank	40.47	63.10			700.5	<.001
Online frequency, mean rank	52.08	49.76			1210.5	.46
Online duration, mean rank	44.54	58.43			920.0	.01
Online activity “information research”, mean rank	55.78	45.51			1011.0	.046

### Confirming the Four-Factor Structure

To ensure that each item loaded on the dimension to which it theoretically belonged, we first conducted an explorative factor analysis (principal axes factor analysis, varimax rotation with Kaiser normalization). The Kaiser-Meyer-Olkin measure of sampling adequacy was .815; the Bartlett’s test of sphericity was significant. The results of the principal axes factor analysis showed the items “because it helps you enjoy your free time,” “so that others don’t make fun of you,” “to be in high spirits,” and “to have more self-confidence” were not strongly related to any dimension. In addition, leaving out these items from the dimension to which they were intended to belong resulted in higher explained variances of the factors and thus in total (Table 4). Consequently, only 16 items (4×4 solution) were included in the final version of the IMQ-A. The wording of all included items is provided in Table 5.

**Table 4:** Factor loadings of the 4 omitted items and coefficients of determination (*R* ^*2*^) of the factors from principal axes analysis following varimax rotation (n=70).

Item	Factor 1: coping	Factor 2: social	Factor 3: enhancement	Factor 4: conformity
Because it helps you to enjoy your free time	.329	–.344	.389	.229
So that others don’t make fun of you		–.336		.360
To be in high spirits	.591	–.293	.412	
To have more self-confidence	.578	–.413		.370
*R* ^*2*^ including the items	29.2	13.6	9.6	6.7
*R* ^*2*^ excluding the items	29.0	15.6	10.2	8.1

The given identifiability (number of estimated parameters is lower than the number of sample moments; 40 <136) justifies the performance of a specified CFA [54]. The CFA of the 16-item IMQ-A yielded significant factor loadings ranging from λ=.22 to λ=.89 (Table 5). The factor coping had the highest item loadings, followed by the factor social. Consequently, these 2 factors also showed the highest internal consistencies. The factor conformity had the lowest loadings and internal consistency. The highest correlation was found between enhancement and coping, the lowest between coping and social. The fit indexes χ^2^_red_, CFI, and RMSEA suggested good model fit (χ^2^_red_=1.5; CFI=.912; RMSEA=.071, 95% CI .045-.094). TLI and SRMR were very close to the recommended thresholds (TLI=.890; SRMR=.088); therefore, they indicated a merely acceptable fit [54-56].

**Table 5:** Item factor loadings, item means, interfactor correlations, and internal consistencies as results of the confirmatory factor analysis to test the 4-factor structure of motives for Internet use.

Items of the IMQ-A		Enhancement	Coping	Social	Conformity	*P*	Mean (SD)
**How often do you go online (*r*):**							
	Because it gives you a pleasant feeling?	.77				<.001	2.60 (1.21)
	Because it is exciting?	.63				<.001	2.35 (1.13)
	To experience a feeling of exaltation?	.69				<.001	1.68 (0.88)
	Simply because it is fun?	.29				.01	3.76 (0.97)
	To forget your worries?		.77			<.001	2.33 (1.14)
	Because it helps you when you feel depressed or irritated?		.74			<.001	2.72 (1.13)
	To cheer yourself up when you are in a bad mood?		.66			<.001	2.87 (1.11)
	To forget about your problems?		.87			<.001	2.40 (1.20)
	To come into contact with others?			.89		<.001	3.51 (1.35)
	Because it is fun to be in contact with others?			.65		<.001	2.86 (1.18)
	To improve your contact with friends and acquaintances?			.82		<.001	3.10 (1.15)
	To share a special occasion with friends?			.55		<.001	2.76 (1.16)
	Because your friends pressurized you to do it?				.22	.08	1.41 (0.70)
	Because you would like to belong to a certain circle of friends?				.75	<.001	1.45 (0.79)
	To be liked by others?				.38	.005	1.52 (0.81)
	To not feel excluded?				.61	<.001	1.61 (0.94)
**Interfactor correlations, *r* (*P*)**							
	Coping×	.66 (<.001)					
	Social×	–.38 (.007)	–.52 (<.001)				
	Conformity×	.32 (.05)	.30 (.04)	.10 (.47)			
Internal consistencies, Cronbach α		.80	.86	.82	.71		

### Concurrent Validity of the IMQ-A

The logistic regression analysis revealed that enhancement and coping but not social and conformity were significantly related to high-risk Internet use (Table 6). The 4 motive dimensions explained approximately 50% of the variance in high-risk Internet use (*R* ^*2*^=49.6%). Testing the relationship between the 4 IMQ-A dimensions and the OSV_K_-S score revealed an effect of enhancement and coping as well as conformity (Table 6), but not of social. The explained variance in the OSV_K_-S score was 33.8%.

**Table 6:** Motives for Internet use as predictors of problematic Internet use and the OSV_K_-S sum score.

Motives	Dysfunctional Internet use? (yes/no)		OSV_K_-S sum score	
	OR (95% CI)	*P*	β^a^	*P*
Social	0.85 (0.77, 1.01)	.06	–.16	.09
Enhancement	1.33 (1.08, 1.64)	.008	.29	.004
Coping	1.19 (1.02, 1.40)	.03	.22	.04
Conformity	1.17 (0.92, 1.48)	.21	.22	.02

^a^ Standardized regression weight (β).

### Motive Ranking Orders

**Figure 1:**
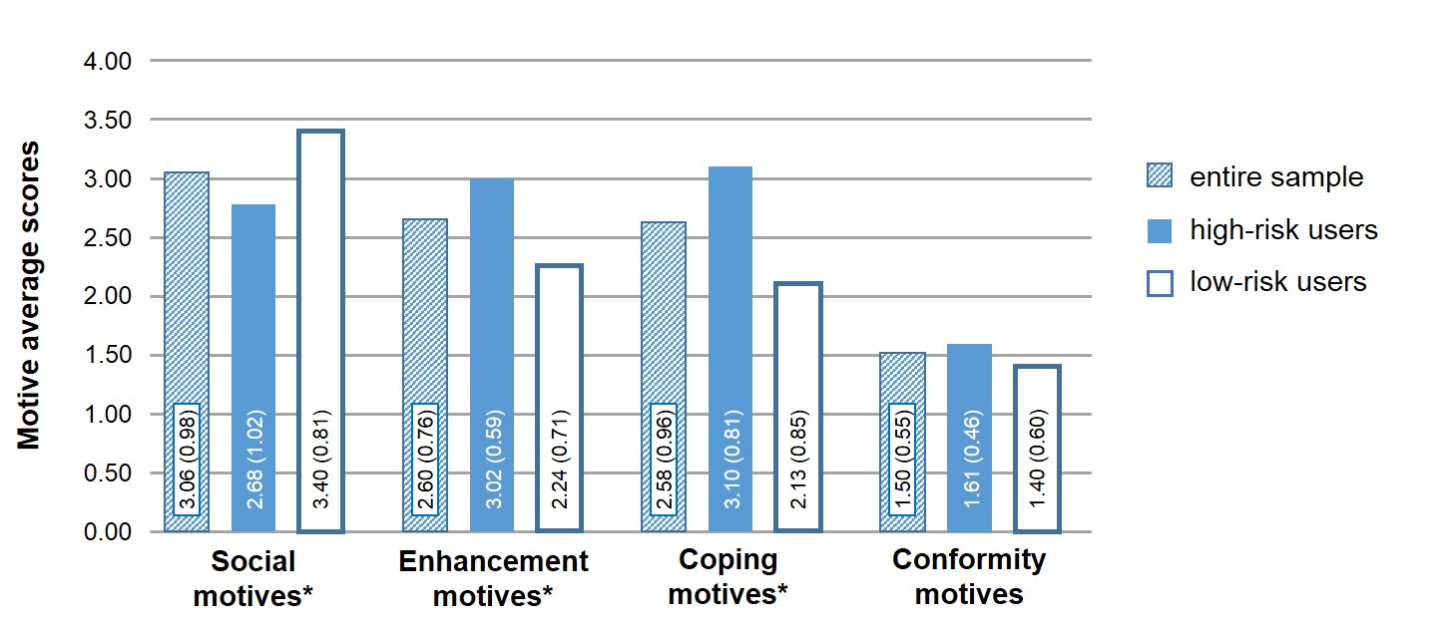
Comparison of the average scores of the 4 motive groups in the total sample and according to risk group. Shown are mean (SD). * indicates *P*<0.001.

In the entire sample, adolescents most frequently accessed the Internet due to social motives followed by enhancement or coping motives (Figure 1). Conformity motives rarely or never applied to participants.

Comparing the motive ranking order in both groups (high-risk and low-risk users), the 2×4 mixed-model ANOVA revealed a group membership (*F*_1, 101_=15.317; *P*<.001) main effect (high risk > low risk) as well as an Internet motive dimension (*F*_2.297, 101_=81.24; *P*<.001) main effect (social > enhancement > coping > conformity). These effects were qualified by a motive × group interaction (*F*_2.297, 101_=27.196, *P*<.001). Post hoc (Bonferroni) tests revealed a significant difference for enhancement, coping, and social, but not for conformity (Figure 1). Members of the high-risk group were predominantly motivated internally in their Internet consumption (coping > enhancement > social > conformity), whereas low-risk users indicated both internal and external motives, but were primarily motivated socially (social > enhancement > coping > conformity).

## Discussion

To provide a basis for early intervention and preventive measures, understanding the motives that drive intensive Internet use to become dysfunctional among adolescents is important. Therefore, the aim of this study was to develop the IMQ-A as a theory-based measurement for identifying the underlying motives for high-risk Internet use.

Because every adolescent uses the Internet daily [3,5-7] and using the Internet is now regarded as the fourth cultural competence, the widespread nature of the Internet is undisputed. Because of this, distinguishing between intensive and dysfunctional adolescent Internet use becomes difficult. Studies regarding 3 other problematic behaviors of adolescents show that motives are a predictor for addictive behavior. Furthermore, motives are comparable across different cultures [41-43,58], which could be explained by the link between motives and personality [22,24-27,58]. As a consequence, developing the IMQ-A based on a questionnaire that had already been developed and validated for different behaviors and cultures not only allows the underlying motives for high-risk Internet use to be identified, but it also offers starting points for developing preventive measures or early intervention programs as well as the chance to compare motivations for different behaviors across different cultures.

Testing the construct validity, the results of the CFA revealed an acceptable, but not outstanding, fit of the motives for Internet use from the 4-factor model. Two factors may be responsible for the comparatively low model fit. First, we used a rather small sample, which turned out to be quite heterogeneous because of the various different recruitment methods. Secondly, IMQ-A items were developed based on an instrument constructed to measure drinking motives. Despite considerable similarities between Internet motives and drinking motives, the item “because your friends pressured you to do it,” for instance, seems to be worded too actively and too strongly for the Internet context, which is reflected by a weak or nonsignificant item loading. Nevertheless, all the other items showed significant loadings on the expected scales. Moreover, at least satisfactory internal consistencies were obtained for all dimensions [53].

The results of the regression analyses support the concurrent validity of the IMQ-A. In accordance with the literature concerning drinking motives [59,60] and gambling motives [41], enhancement and coping predicted the 2 criteria of dysfunctional Internet use (prediction of high-risk users and higher OSV_K_-S score). The consistency of results across the 2 indicators highlights the robustness of the findings and indicates that accessing the Internet frequently for internal emotion-regulation motives is more dysfunctional than social Internet use, which appears more recreational. Our findings showed that persons characterized as low-risk users seem to use the Internet mainly to meet social needs and maintain contacts. Consequently, it can be supposed that the Internet (as is the case with alcohol consumption or gambling) is also used as a method for regulating emotions, and when this motivation comes into play, Internet consumption may exceed the useful aspects.

Concluding from the results of the CFA and the regression analyses, construct and concurrent validity can be regarded as given. Therefore, it can be assumed that motives of adolescent Internet consumption can be measured using the developed questionnaire. By implication, the respondents’ answers reveal that they use the Internet both for information research and reasons of social identity and integrity as well as for entertainment as defined by McQuail [19,20].

The results regarding the motive ranking suggest that adolescents access the Internet primarily for positive motives (enhancement and social), whereas negative motives (coping and confirmatory) seem to have a minor impact. Interestingly, these results are consistent with the drinking motives literature [38,40,60,61]. Further analogies to the field of drinking motives can be drawn [40] based on the results of group-specific analysis. Thus, high-risk users mainly accessed the Internet for internal motives, whereas low-risk users indicated both internal and external motives, but were primarily motivated socially.

Although the presented results demonstrate the psychometric qualities of the IMQ-A to assess adolescent motives for accessing the Internet, it is likely that there are other motives for accessing the Internet besides affective change, such as gaming or knowledge acquisition [3-5,22-25]. Another limitation is the small sample size and the fact that the current sample is not representative of all Internet users in this age group. For example, compared with other studies [12-17], the prevalence of dysfunctional Internet users (46.5%) indicates an overrepresentation of problematic users. One explanation could be the Web-based data collection method as well as the fact that 2 OSV_K_-S categories were rolled into 1 (problematic and addicted to high risk).

Consequently, we recommend further validation of the IMQ-A with larger sample sizes, possibly including other cultures or parts of Germany. Moreover, in the presented survey, the IMQ-A was applied to a nonclinical sample. For this reason, it remains unclear to what extent the questionnaire can be used in a clinical sample. Future longitudinal studies are recommended to further examine the predictive validity. In this way, tests could be conducted to see whether the IMQ-A predicts future Internet use behavior among adolescents.

Despite the described limitations, the IMQ-A appears to be a valid and reliable instrument to assess motives related to adolescent Internet use. This questionnaire could serve as a basis on which to develop preventive measures or early intervention programs dealing especially with internal motives of Internet consumption.

In the clinical field, the IMQ-A can help to identify the motives of dysfunctional Internet consumption and establish individual intervention aspects for developing alternative coping strategies for coping users, for example.

## Multimedia Appendix 1

The Internet Motive Questionnaire for Adolescents (IMQ-A; German version).

## Multimedia Appendix 2

The Internet Motive Questionnaire for Adolescents (IMQ-A; English version).

## Abbreviations

ANOVA: analysis of variance
CFA: confirmatory factor analysis
CFI: comparative fit index
χ2red: reduced chi-square (chi-square divided by degrees of freedom)
CSV-R: screening scale for computer gaming behavior (“Screener zum Computerspielverhalten”)
DMQ-R: Drinking Motives Questionnaire Revised
DSM: Diagnostic and Statistical Manual of Mental Disorders
IAT: Internet Addiction Test
ICD: International Statistical Classification of Diseases and Related Health Problems
IMQ-A: Internet Motive Questionnaire for Adolescents
MOGQ: Motives for Online Gaming Questionnaire
OSVK-S: Screening measure for online addiction behavior in children and adolescents (“Online-Suchtverhalten-Skala”)
RMSEA: root mean square error of approximation
SRMR: standardized root mean squared residual
TLI: Tucker-Lewis index
