# Supplementary material for: Identifying Problematic Internet Users: Development and Validation of the Internet Motive Questionnaire for Adolescents (IMQ-A)
Source: J Med Internet Res. 2014 Oct 9;16(10):e230. doi: 10.2196/jmir.3398 (PMC4210952; doi:10.2196/jmir.3398)
Supplement: Supplementary file 1 [file jmir_v16i10e230_app1.pdf]

Internet Motive Questionnaire for Adolescents (IMQ$_A$)
# - Originalversion Deutsch -

C. Bischof-Kastner, E. Kuntsche, J. Wolstein

Denke an die Gelegenheiten, in denen du in den letzten 12 Monaten im Internet online warst, wie oft bist du online...

Kreuze in jeder Zeile ein Kästchen an.

		(fast) nie	manchmal	öfters	meistens	(fast) immer
a)	um deine Sorgen zu vergessen?	☐	☐	☐	☐	☐
b)	weil dich deine Freunde/-innen dazu gedrängt haben?	☐	☐	☐	☐	☐
c)	weil es dir hilft, wenn du niedergeschlagen oder gereizt bist?	☐	☐	☐	☐	☐
d)	um mit anderen in Kontakt zu kommen?	☐	☐	☐	☐	☐
e)	um dich aufzumuntern, wenn du in schlechter Stimmung bist?	☐	☐	☐	☐	☐
f)	weil dir das ein gutes Gefühl gibt?	☐	☐	☐	☐	☐
g)	weil es aufregend ist?	☐	☐	☐	☐	☐
h)	um eine Art Hochgefühl zu erleben?	☐	☐	☐	☐	☐
i)	weil es dann spaßiger ist, wenn du mit anderen in Kontakt bist?	☐	☐	☐	☐	☐
j)	weil du gerne zu einer bestimmten Clique gehören möchtest?	☐	☐	☐	☐	☐
k)	um den Kontakt zu Freunden/-innen oder Bekannten zu verbessern?	☐	☐	☐	☐	☐
l)	um ein besonderes Ereignis mit Freunden/-innen zu teilen?	☐	☐	☐	☐	☐
m)	um deine Probleme zu vergessen?	☐	☐	☐	☐	☐
n)	weil es einfach Spaß macht?	☐	☐	☐	☐	☐
o)	um von anderen gemocht zu werden?	☐	☐	☐	☐	☐
p)	um dich nicht ausgeschlossen zu fühlen?	☐	☐	☐	☐	☐
		1	2	3	4	5
