# Supplementary material for: Identifying Problematic Internet Users: Development and Validation of the Internet Motive Questionnaire for Adolescents (IMQ-A)
Source: J Med Internet Res. 2014 Oct 9;16(10):e230. doi: 10.2196/jmir.3398 (PMC4210952; doi:10.2196/jmir.3398)
Supplement: Supplementary file 2 [file jmir_v16i10e230_app2.pdf]

Internet Motive Questionnaire for Adolescents (IMQA)
# - English Version -

C. Bischof-Kastner, E. Kuntsche, J. Wolstein

Think of all the times you have been online during the last 12 months, how often do you go online...

Please tick one box in every line.

		(almost) never	some of the time	half of the time	most of the time	(almost) always
a)	to forget your worries?	☐	☐	☐	☐	☐
b)	because your friends pressurized you to do it?	☐	☐	☐	☐	☐
c)	because it helps you when you feel depressed or irritated?	☐	☐	☐	☐	☐
d)	to come into contact with others?	☐	☐	☐	☐	☐
e)	to cheer yourself up when you are in a bad mood?	☐	☐	☐	☐	☐
f)	because it gives you a pleasant feeling?	☐	☐	☐	☐	☐
g)	because it is exciting?	☐	☐	☐	☐	☐
h)	to experience a feeling of exaltation?	☐	☐	☐	☐	☐
i)	because it is fun to be in contact with others?	☐	☐	☐	☐	☐
j)	because you would like to belong to a certain circle of friends?	☐	☐	☐	☐	☐
k)	to improve your contact with friends and acquaintances?	☐	☐	☐	☐	☐
l)	to share a special occasion with friends?	☐	☐	☐	☐	☐
m)	to forget about your problems?	☐	☐	☐	☐	☐
n)	simply because it is fun?	☐	☐	☐	☐	☐
o)	to be liked by others?	☐	☐	☐	☐	☐
p)	to not feel excluded?	☐	☐	☐	☐	☐
		1	2	3	4	5
